# Adenoviruses in Avian Hosts: Recent Discoveries Shed New Light on Adenovirus Diversity and Evolution

**DOI:** 10.3390/v14081767

**Published:** 2022-08-13

**Authors:** Ajani Athukorala, Karla J. Helbig, Brian P. Mcsharry, Jade K. Forwood, Subir Sarker

**Affiliations:** 1 Department of Microbiology, Anatomy, Physiology and Pharmacology, School of Life Sciences, La Trobe University, Melbourne, VIC 3086, Australia; 2 School of Dentistry and Medical Sciences Biomedical Sciences, Charles Sturt University, Wagga Wagga, NSW 2678, Australia

**Keywords:** adenovirus, avian adenoviruses, *Atadenovirus*, *Siadenovirus*, *Aviadenovirus*, coevolution, genetic diversity, host-switching

## Abstract

While adenoviruses cause infections in a wide range of vertebrates, members of the genus *Atadenovirus*, *Siadenovirus*, and *Aviadenovirus* predominantly infect avian hosts. Several recent studies on avian adenoviruses have encouraged us to re-visit previously proposed adenovirus evolutionary concepts. Complete genomes and partial DNA polymerase sequences of avian adenoviruses were extracted from NCBI and analysed using various software. Genomic analyses and constructed phylogenetic trees identified the atadenovirus origin from an Australian native passerine bird in contrast to the previously established reptilian origin. In addition, we demonstrated that the theories on higher AT content in atadenoviruses are no longer accurate and cannot be considered as a species demarcation criterion for the genus *Atadenovirus*. Phylogenetic reconstruction further emphasised the need to reconsider siadenovirus origin, and we recommend extended studies on avian adenoviruses in wild birds to provide finer evolutionary resolution.

## 1. Introduction

Adenoviruses are nonenveloped double-stranded DNA viruses with a linear genome that ranges from about 25 kb to 45 kb [1,2]. The family *Adenoviridae* occurs within the order *Rowavirales*, and there are 87 ICTV-accepted adenovirus species, of which many are fully sequenced [3,4,5]. A large number of partially sequenced adenoviruses are also available, which typically include a portion of the DNA polymerase or hexon encoding-genes, and these are not yet classified into species. Adenoviruses have been divided into six genera,

*Aviadenovirus*, *Atadenovirus*, *Siadenovirus*, *Mastadenovirus*, *Ichtadenovirus*, and *Testadenovirus* [3,6,7,8,9,10]. Within genera, a species could be defined as having > 10–15% phylogenetic distance based on distance matrix analysis of DNA polymerase amino acid sequence or based on the nucleotide composition [3]. Moreover, in humans and domestic animals, there are many genetic variants and serological variants found within species that can vary in pathogenicity.

Adenoviruses likely have a similar structural morphology [11], but the terminal genomic contents differ between viruses in various genera [12]. Based on the times of post-infection, transcription, and generating transcripts, genes are classified into early, intermediate, or late regions. Early genes ensure efficient viral DNA replication and the subsequent late gene expression, while the intermediate IVa2 gene acts as a transcriptional activator and stimulates late gene transcription. Late genes encode both structural and non-structural proteins involved in viral capsid formation, DNA encapsidation, and maturation. Adenovirus virions have three major capsid proteins; hexon (II), penton base (III), and fibre (IV); four minor proteins (IIIa, VI, VIII, and IX); and six other proteins, (V, VII, μ, IVa2, terminal protein, and protease) [1,13,14]. However, notable differences in gene content have been found in the left and right flanking regions, especially the right-hand end of the genome [15], and several studies based on comparative genomic analyses have further confirmed that the right genomic end has the highest variability with unique gene content among aviadenovirus species [15,16,17,18,19].

Enhancement of molecular detection technologies has led to an exponential growth in understanding of adenovirus genomics. Virus characterisation that relies on initial isolation of virions in cell-culture systems followed by electron microscopy and immune-based methods for virus characterisation requires viruses to grow in different cell culture systems, with such systems lacking for many avian adenoviral species. Early Sanger sequencing attempts managed to overcome the limitations of the lack of an appropriate culture system for in vitro virus propagation, and the recent advent of next-generation sequencing technology has led to the exponential growth in adenovirus sequencing information [8,20]. In this study, we described the progression of adenovirus genomics, including genome-wide comparisons of adenovirus sequences, viewpoints of adenovirus evolution based on recent research data, and potential future directions for the field, with an emphasis on avian adenoviruses.

## 2. Materials and Methods

### 2.1. Retrieval of Adenovirus Sequences and Alignment

Nucleotide sequences of adenoviruses that have been accepted as species by ICTV and that were publicly available by January 2021 were extracted from the NCBI database, and amino acid sequences were extracted using Geneious Prime^®^ (version 2022.1.1, Biomatters, Auckland, New Zealand). Sequences were selected representing all strains, lineages, and geographical regions, avoiding duplication and aligned using MAFFT (version 7.388) [21] in Geneious Prime^®^ (version 2022.1.1, Biomatters, Auckland, New Zealand). Nucleotide sequences of the cytochrome c oxidase subunit I (COX1) gene of selected host species were downloaded from the NCBI database. Sequence information of adenoviruses and host organisms used in this work are listed in Supplementary Table S1. 

### 2.2. Phylogenetic Tree Construction and Evolutionary Analyses

Maximum likelihood (ML) trees were constructed using amino acid sequences of the complete DNA-dependent DNA polymerase gene from the selected adenoviruses. Protein sequences of DNA-dependent DNA polymerase gene were aligned with MAFTT (version 7.388) [21] in Geneious Prime^®^ (version 2022.1.1, Biomatters, Auckland, New Zealand) under the BLOSUM62 scoring matrix. Pairwise similarities were computed for the corresponding alignments using Geneious Prime^®^ (version 2022.1.1, Biomatters, Auckland, New Zealand). (Supplementary Table S2), and the unrooted ML trees were constructed with PhyML [22], and 500 bootstrap resampling was chosen. A model test was performed using CLC Genomics Workbench (version 9.0.1, CLC bio, a QIAGEN Company, Prismet, Aarhus, Denmark) to determine the best-fit model. Developed virus phylogeny using complete DNA-dependent DNA polymerase genes (Supplementary Figure S2) and host phylogeny based on cytochrome c oxidase subunit I gene sequences (Supplementary Figure S3) were used to generate tanglegram in Dendroscope (version 3.8.2, Free Software Foundation, Boston, MA, USA) [23]. Bootstrap network generated using SplitsTree 4.15.1 under 1000 replicates and partial DNA polymerase gene amino acid sequences (Supplementary Table S3) from representative adenoviruses [2,9,16,17,18,24,25,26,27,28,29,30,31,32,33,34,35,36,37,38,39,40,41,42], including adenovirus sequences reported in three recent independent studies [10,43,44]. In addition, an ML tree was generated using concatenated amino acid sequences of four major structural and functional genes: complete DNA-dependent DNA polymerase, penton, pTP and hexon. 

## 3. Results and Discussion

Adenoviruses detected in avian host described by genera.

### 3.1. Atadenovirus

#### 3.1.1. Genome Content

**Figure 1:**
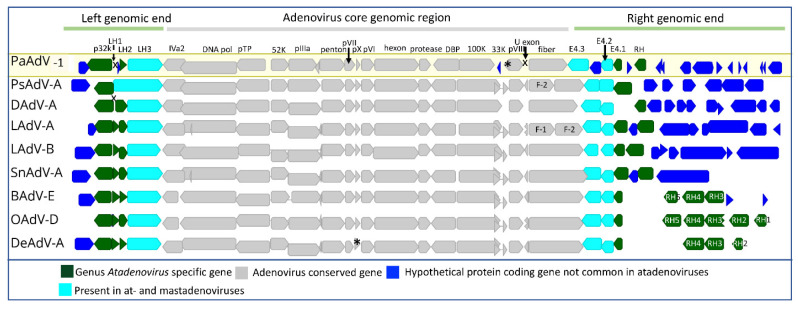
Schematic diagram of atadenovirus genome comparison. Complete atadenovirus genomes were extracted from the following: NCBI (passerine adenovirus 1 (PaAdV-1), GenBank Ac no. MT674683; *Psittacine atadenovirus A* (PsAdV-A), GenBank Ac no. KJ675568; *Duck adenovirus A* (DAdV-A), GenBank Ac no. KF286430; *Lizard atadenovirus A* (LadV-A), GenBank Ac no. KJ156523; *Lizard atadenovirus B* (LadV-B), GenBank Ac no. MT050041; *Snake atadenovirus B* (SnAdV-B), GenBank Ac no. DQ106414; *Bovine atadenovirus B* (BadV-B), GenBank Ac no. JQ345700; *Ovine atadenovirus A* (OadV-B), GenBank Ac no. NC_004037 and *Deer atadenovirus B* (DeAdV-B), GenBank Ac no. MK343439) and they were aligned with MAFTT (version 7.388) [21] in Geneious Prime^®^ (version 2022.1.1, Biomatters, New Zealand). Adenovirus conserved genes, genus-specific genes, and species-specific hypothetical open reading frames are presented in different colours. The significance of missing conserved genes in the presented genomes is marked with X, and genes present, but not annotated are represented with a star (*). F-1 and F-2 are the abbreviations for fibre 1 and 2, respectively.

Of the atadenoviruses that have been fully sequenced, genomes are in the range of 27–39 kb [8], with the fully sequenced virus from a spinebill (passerine adenovirus 1) having the largest genome (39,664 bp) [16] and *Snake atadenovirus A* from corn snake having the smallest genome (27,751 bp) [45]. Atadenoviruses exhibit a similar degree of diversity to other adenovirus genera, and an approximately 50% sequence homology within the conserved DNA polymerase gene between distantly related atadenoviruses can be observed (Supplementary Table S2). The complement of core genes found in atadenoviruses is also similar to the other adenoviruses (Figure 1). Notable exceptions include the absence of a U exon gene in passerine adenovirus 1 (PaAdV-1, Accession number MT674683) genome. Additionally, *Lizard atadenovirus A* (LAdV-A) isolated from a Mexican beaded lizard and *Psittacine atadenovirus A* (PsAdV-A) identified during an outbreak possess a unique fibre gene arrangement for atadenoviruses, comprising two fibre genes, encoding one short and one long fibre protein [35,45,46].

Atadenoviruses also contains three unique genes, LH3, E4.2, and E4.3, which are found to be the distant homologs of genes encoding E1b 55k and E4 34 K in mastadenoviruses, sharing the equivalent genome positions and similar functions [5]. The LH3 homolog, 55k, interferes with the host’s ability to degrade the viral DNA and inhibit DNA damage responses [47,48]. LH3 also shows a similar architectural role to the mastadenovirus-specific protein IX as a cement protein, important in outer capsid stability [49,50]. So, it can be speculated that diverse genes found in this variable region in atadenovirus genome ends could play functionally important roles in regulating host responses.

Like other adenovirus genera, atadenoviruses have unique genes in the left hand (LH) and right hand (RH) portions of their genomes. The right-hand region of the atadenovirus family contains most of the hypothetical open reading frames (ORFs) in comparison to the left genomic end (Figure 1). RH0 to RH6 genes in the right-hand region are also unique to atadenoviruses, but not common to all atadenovirus species [5]. All atadenoviruses contain the genus-specific structural protein p32K followed by LH1, LH2, and LH3 genes, with some exceptions. The LH1 gene is absent in *Duck atadenovirus A* and passerine adenovirus 1 genomes (Figure 1), while both LH1 and LH2 genes are missing in the *Psittacine atadenovirus A* genome (Figure 1). As mentioned above, the functions of the products of the open reading frames are predicted in some instances, but a significant number of these remain uncharacterised. Of these open reading frame products, some appear to be captured from other organisms, including bacteria, phages, and other virus hosts [50]. Some of these are unique to the viruses that have been sequenced, and some are shared with other more closely related viruses; For example, the LH3/E1b 55K-like β helix proteins shares homology with specific bacteria and phages, indicating that these genes may have transferred to a common ancestor of at- and mastadenoviruses [50]. 

#### 3.1.2. Evolution 

The discovery of novel full-length and partial atadenovirus sequences in birds and reptiles presents some inconsistencies compared to our current understanding of atadenovirus evolution. Atadenoviruses were initially named because the first three sequenced viruses had AT-rich genomes [51,52]. Based on this early sequence data, it appeared that they originated in reptiles with possible host switching events resulting in infections in birds and several ruminant species (Supplementary Figure S1). More recently with the discovery of enhanced numbers of atadenovirus strains from reptiles, birds, and mammals; other possible routes of adenovirus evolution have been hypothesized, including one suggesting that they first evolved in passerine birds [16]. 

**Figure 2:**
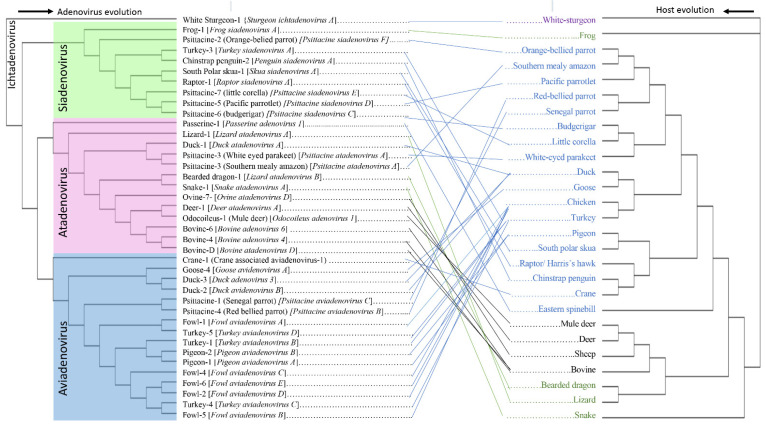
An illustration showing the evolution of three major adenovirus genera with their respective host species denoting the adenovirus cross-species transmission to completely different hosts more than coevolution with host species. Phylogenetic trees of adenoviruses using DNA polymerase sequences and respective hosts using cytochrome c oxidase subunit 1 were generated and used to develop tanglegram in Dendroscope (version 3.8.2, Free Software Foundation, Boston, Massachusetts, USA). Bootstrap support for individual virus and host trees are shown in Supplementary Figures S2 and S3. The labels at branch tips refer to the original adenovirus species name, and lines indicate the association with the host organism. The genus *Ichtadenovirus* is left as an outgroup, and the genus *Mastadenovirus* and *Testadenovirus* is not presented. Three major adenovirus genera are considered, and bird (blue), mammal (black), and reptiles/amphibians (green) hosts are shown in different colours.

Here, we present a rigorous phylogenetic analysis using the adenovirus DNA polymerase gene sequences, to provide the best estimate of atadenovirus phylogeny and potential evolutionary relationships. Phylogenetic analysis demonstrated that atadenoviruses isolated from birds, squamates, and ruminants are represented by three different clades (Figure 2). This phylogenetic tree was generated using the host species of the individual adenoviruses and pairing with matching leave ends of the host phylogeny to understand the evolutionary relationship of adenoviruses along with their respective host species. Paired events occurred in response to the virus and host coevolution, while host switches to distant relatives are represented by the crossing lines [53]. Matching speciation events between *Psittacine atadenovirus A* and the host, white-eyed parakeet exhibited the strict pattern of co-speciation of atadenovirus with their host organism. Additionally, some atadenoviruses infect closely related hosts, while others display a tendency to jump to a distant relative and to appear in a different clade [8]. The basal clade and ancestral host Australian passerine species, the Eastern spinebill (*Acanthorhynchus tenuirostris*), from which psittacines and a single Anseriforme virus were first evolved, now contains multiple virus variants [16]. Reptile infection appears to have resulted from a host switch from birds to reptiles and ruminant adenoviruses appear to have resulted in a second host switch from an ancestral reptile adenovirus. Phylogenetic reconstruction was also performed using concatenated protein sequences of two structural proteins, penton and hexon, and two non-structural proteins, DNA polymerase and pTP (Supplementary Figure S1), which confirmed a similar pattern of atadenovirus evolution and was also shown previously by Athukorala et al. 2020 [16]. 

In addition to the complete viral genome sequences of atadenoviruses, and more generally adenoviruses, many partial (typically encoding short sequences of 90 amino acids) novel adenovirus DNA polymerase sequences have been reported in birds and reptiles. Phylogenetic trees deduced from partial DNA polymerase sequences by Rinder et al. (2020) [44] with 25 putative adenoviruses identified from European passeriform birds show a diverse range of atadenovirus hosts with possible avian atadenovirus origin. The more descriptive phylogenetic analysis performed by Vaz et al. (2020) [10] resulted in four different adenovirus lineages with two sublineages, predominantly grouping lineage 1 (passerines and psittacines), 2 (ruminants), 3A (snakes and lizards), 3B (reptiles and different bird species), and 4 (passerines). Based on new sequence data from birds and reptiles, it is clear that there is extensive, previously unrecognized atadenovirus diversity and that some hosts may harbour multiple atadenovirus species.

**Figure 3:**
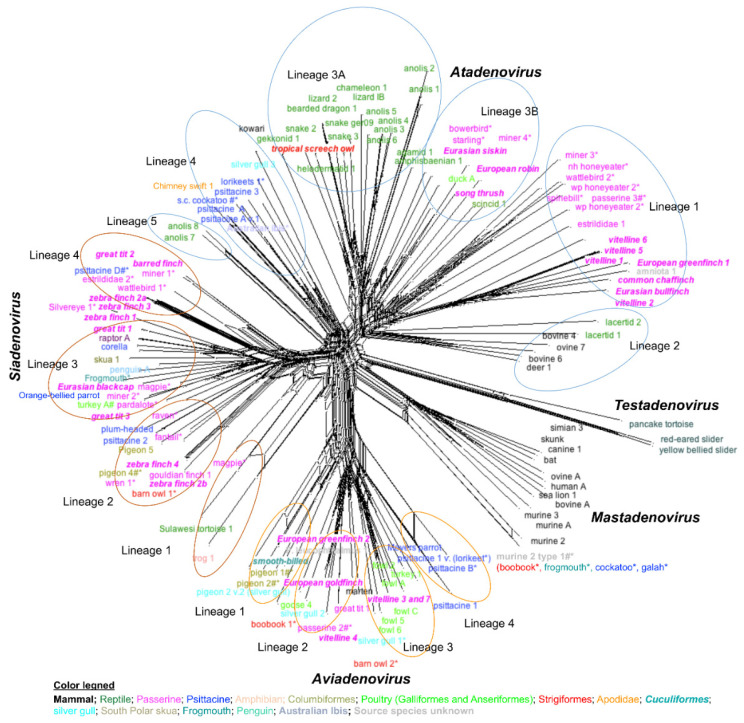
Bootstrap network analysis using partial DNA polymerase gene amino acid sequences. Bootstrap values that exceeded 80% are highlighted with line weight as generated by SplitsTree software. Groups of host species were coloured as shown in the colour legend. Murine adenovirus 2 variant 1 was found in the four species of birds in parenthesis; however, it is suspected to be of murine origin. Virus sequences identified in the study conducted by Vaz et al. (2020) [10] were shown with an asterisk (*), whereas adenoviruses identified with a hashtag (#) were found in more than one species of bird, either in the study by Vaz et al. (2020) [10], and/or one or more studies by others [2,9,16,17,18,24,25,26,27,28,29,30,31,32,33,34,35,36,37,38,39,40,41,42]. Recent adenovirus sequences reported from Europe [44] and South America [43] were also included and highlighted with a bold and italic font. Lineages are designated according to Vaz et al. (2020) [10].

As these sequences are so short, it is not possible to use them to produce a robust phylogenic tree with the support of deep roots. However, it is possible to use these sequences to appreciate the complexity/diversity of the atadenoviruses and roughly predict the relationships between them using alternate methods. In Figure 3, an estimate of the increasingly complex structure of the *Adenoviridae* has been demonstrated. By including all currently known atadenovirus sequences and combining the recent findings [10,44], the unrooted tree generated in Figure 3 demonstrated five different lineages (lineages 1 to 5) within the genus *Atadenovirus*. Lineage 3 could additionally be subdivided into sublineages 3A and 3B (Figure 3). Avian hosts dominate lineages 1, 3B, and 4, while ruminants and reptiles exist predominantly in lineages 2 and 3A, respectively. Lineage 1 comprised passerine adenoviruses derived from Europe, Australia, and some African species kept in zoological gardens, and it contained two different non-overlapped subclades (Figure 3). Thus, the phylogeny indicates the coevolution of atadenoviruses in lineage 1 and their respective avian passerine hosts over time.

Under the genus *Atadenovirus*, reptiles were appearing in three different lineages, Lineage 2, 3A, and 5 and were dominant in Lineage 3A with several snakes and lizards, usually native to different landmasses in the world. With the lack of sufficient sequence information from wild species, it is difficult to establish a direct conclusion on their evolutionary relationships based mostly on captive animals. Additionally, only limited conclusions can be drawn from the sequences found in lineage 3B, due to the fact that the majority of viruses are derived from passerine birds; therefore, it appears that this is a passerine-adapted clade. However, sequences in this clade come from both Australian and European species with no evidence of coevolution in these geographically separated species. It is difficult to conclude with inadequate adenovirus sequence data from passeriformes, but the phylogenetic reconstruction performed in this study, represents the possible introduction of European adenoviruses into native Australian species, where there is transmission of viruses from invasive species to native species and vice versa [10].

In addition to major host switching events hypothesized to have resulted in the reptile and ruminant clades, host switching has also been seen to occur within clades. This includes the appearance of the distantly related Rainbow lorikeet, silver gull, and Australian ibis adenoviruses among psittacines in lineage 4, and duck adenovirus A forming a clade with passerines in lineage 3B (Figure 3) [10]. Other possible host switching events may include the tropical screech owl and the kowari (Figure 3). Tropical screech owl adenovirus 1 was found in the faeces of a tropical screech owl; therefore, the sequence could either have originated from the owl itself or could have been a virus that infected a reptilian prey species of this owl [43]. Tropical screech owl adenovirus 1 clusters with the reptile viruses in clade 3. Seeming less likely, it could represent a host switching event where the tropical screech owl was originally infected with a reptile virus. However, the mammalian clade (kowari) [27] amongst psittacine birds is a significant cross-species transmission of atadenoviruses rather than coevolution. The search for food and habitat sharing may provide the likely opportunities for atadenoviruses to switch their hosts. That this virus was detected in the tissues of an animal that had lesions consistent with an adenovirus infection [8,27] might indicate it has bird origin and switched to a mammalian host. Lastly, Amniota virus found in Brazil in captive animals appears in Lineage 1, possibly from the pooled faecal samples from birds and a mammal (Figure 3) [10].

Originally, it was proposed that the atadenoviruses were composed of adenoviruses that had an A + T bias and that this might be indicative of recent host switching events. This theory was based on early available genome selections with higher A + T content [54], and the genus *Atadenovirus* was originally named reflecting this exceptional characteristic of strikingly high A + T (>60%) content detected in genomes of early atadenoviruses members: ruminants, birds, and a marsupial. However, subsequent sequences from birds, snake, and lizard sequences identified a balanced G + C content in contrast to initially detected atadenovirus genomes [8,10,11,16,35,40] and evidenced that low G + C content is not a consistent criterion to be considered for the Atadenovirus genus. A recent study on bird adenoviruses conducted by Vaz et al. (2020) [10] highlighted that the G + C percentage can vary from 31 to 67%, providing an example of variation in G + C content in two clusters of viruses originated from passerine birds in atadenovirus lineage 1. This trend seems to continue with the analysis including European passerines in this study. Vaz et al. (2020) [10] recommended that this criterion can be considered to distinguish between relatively closely related sequences that originate from similar species of birds. Importantly, Athukorala et al. (2020) [16], shows the likely evolutionary trend of atadenovirus from a passerine adenovirus 1 and its kin, and the parrot adenovirus 3 and its kin, to an increased A + T content by emerging into Anseriformes, squamates, with the highest being found in the ruminant viruses.

Information is lacking on atadenovirus genome sequences from other parts of the world including Africa, North America, South America, and Asia, with most of the available data generated based on Australian and European bird species. A better understanding of sequence data, especially from passerines from these unattended regions, would be highly beneficial to clarify atadenovirus evolution.

### 3.2. Siadenovirus

#### 3.2.1. Genome Content

The *Siadenovirus* genus represents the shortest known genomes in the adenovirus family, ranging approximately 26–27 kb in size with a G + C content of 34.9 to 38.5%, and encoding small inverted terminal repeat (ITR) regions usually around 29–39 bp [2,5,8,55,56,57]. The sequence diversity within the genus *Siadenovirus* is also recorded to be as high as 35% (Supplementary Table S2) based on the DNA polymerase gene. Except for the central conserved gene cassette, the genomic organisation of siadenoviruses is distinct from that of other genera.

**Figure 4:**
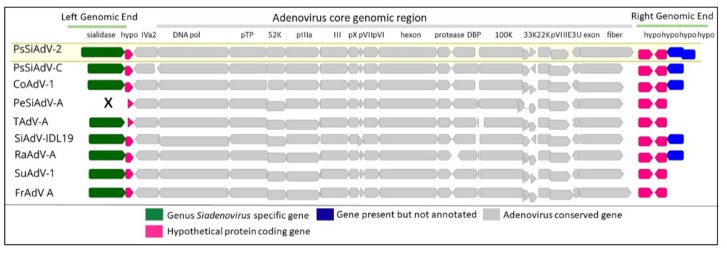
Schematic representation of siadenovirus genome comparison. Complete siadenovirus genomes were extracted from the following: NCBI (*Psittacine siadenovirus F* (PsSiAdV-F), GenBank Ac no. MW365934; *Psittacine siadenovirus C* (PsSiAdV-C), GenBank Ac no. MN687905; *Corella adenovirus A* (CoAdV-A), GenBank Ac no. MK227353; *Penguin siadenovirus A* (PeSiAdV-A), GenBank Ac no. KP144329; *Turkey adenovirus A* (TAdV-A), GenBank Ac no. AC_000016; Siadenovirus—strain IDL 19 (SiAdV-IDL 19), GenBank Ac no. MK695679; *Raptor adenovirus A* (RaAdV-A), GenBank Ac no. EU715130; *Skua adenovirus B* (SuAdV-B), GenBank Ac no. HM585353 and *Frog siadenovirus A* (FrAdV-A), GenBank Ac no. NC_002501); they were aligned with MAFTT (version 7.388) [21] in Geneious Prime^®^ (version 2022.1.1, Biomatters, New Zealand). Adenovirus conserved genes, genus-specific sialidase, genes common and species-specific hypothetical proteins were presented in different colours. The significance of missing conserved gene is marked with X.

Most of the siadenoviruses share common adenovirus core genes and genus-specific genes in their right and left termini; however, there are a few exceptions. Within the left-hand region of *Penguin siadenovirus A*, the sialidase gene is absent (Figure 4) [58]. This gene is positioned within the left-hand end between the ITR region and a common protein coding region and encodes a unique putative sialidase protein (neuraminidase) which underpins the genus name [55]. This flanking ORF (Figure 4; pink colour ORF in Left genomic end) in some species (FrAdV-A and TAdV-A) is predicted to encode highly hydrophobic proteins. In addition, the right genomic end of all known complete siadenovirus genomes were also shown to contain two hypothetical protein-coding genes (ORF7 and -8) (Figure 4). These conserved hypothetical genes may have a genus-specific function, which is yet to be identified. 

#### 3.2.2. Evolution

Siadenoviruses are proposed to have an amphibian origin, but there is little evidence to support this conclusion. Genus *Siadenoviruses* consists of eight accepted species: *Frog siadenovirus A*, *Great*-*tit siadenovirus A*, *Penguin siadenovirus A*, *Raptor siadenovirus A*, *Psittacine siadenovirus D*, *Psittacine siadenovirus E*, *Skua siadenovirus A* and *Turkey siadenovirus A* [5,9,55,56,58,59]. *Frog siadenovirus A* was reported to be isolated from a cell line of reptilian origin [8,24] and it is the basal to all siadenoviruses (Supplementary Figure S1) that were isolated from avian hosts. This significant crossing between distantly related frog and avian hosts suggests the major host switching event occurred in early siadenovirus evolution (Figure 2). Siadenovirus that infects frogs crossed the genomic barriers to infect psittacine birds (*Psittacine siadenovirus F*) [60,61] which was recently identified as the closest relative to frog adenovirus that then evolved into a few distinct subclades [60] (Supplementary Figure S1). However, it remains a mystery that no other siadenovirus have been detected in amphibians (frogs or salamanders) to date, even after several studies [8,62], and therefore, the hypothesis of the amphibian origin of siadenovirus remains to be proven [8]. *Psittacine siadenovirus F* appeared to coevolve with their host parrots, but several other host jumps occurred afterward could account for the detection of siadenoviruses in diverse avian groups such as the penguin, raptors, and a galliform (Figure 2 and Supplementary Figure S1). 

Based on the bootstrap network analysis using the available partial DNA polymerase gene sequences of known adenoviruses, we demonstrate a higher diversity of siadenoviruses. As shown in Figure 3, four lineages within the genus Siadenovirus were identified. Lineage 1 comprises viral sequences from a Sulawesi tortoise, frog, and a passerine bird with distantly related subclades. Additionally, the siadenovirus sequence from a Magpie shows such close relatedness to tortoise, that it can only be explained by a significant host switch, possibly from birds to reptiles (Figure 3). It was also observed that Australian and European passerines solely formed lineage 4, but also dominated the other two lineages; lineages 2 and 3 compromised a range of diverse bird hosts including psittacine, Columbiformes, poultry, and Strigiformes (Figure 3). Detection of certain different siadenoviruses in the same host and emerging into phylogenetically distant lineages from each other highlight that multiple possible host-switching events occurred crossing their species boundaries within the family. However, we need more sequence information for a better resolution of the species evolution in the genus *Siadenovirus*.

### 3.3. Aviadenovirus

#### 3.3.1. Molecular Structure

**Figure 5:**
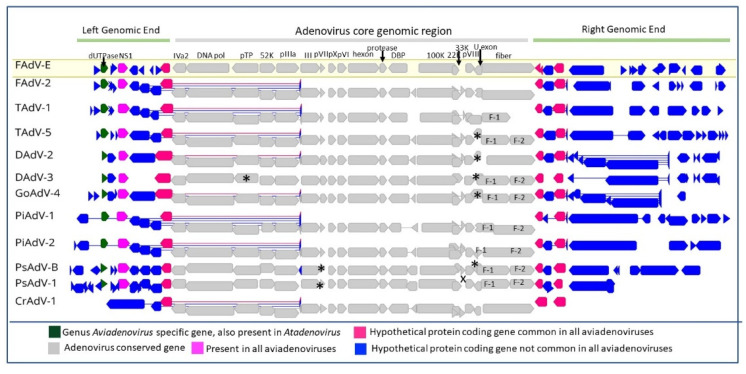
Schematic illustration of aviadenovirus genome comparison. Complete aviadenovirus genomes were extracted from the following: NCBI (*Fowl aviadenovirus E* (FAdV-E), GenBank Ac No. KT862808; *Fowl aviadenovirus D* (FAdV-D), GenBank Ac No. KT862805; *Turkey aviadenovirus B* (TAdV-B), GenBank Ac No. GU936707; *Turkey aviadenovirus D* (TAdV-D), GenBank Ac No. KF477313; *Duck aviadenovirus B* (DAdV-B), GenBank Ac No. KJ469653; *Duck adenovirus 3* (DAdV-3), GenBank Ac No. MW677606; *Goose aviadenovirus A* (GoAdV-A), GenBank Ac No. JF510462; *Pigeon aviadenovirus A* (PiAdV-A), GenBank Ac No. MW286325; *Pigeon aviadenovirus B* (PiAdV-B), GenBank Ac No. KX121164; *Psittacine aviadenovirus B* (PsAdV-B), GenBank Ac No. KX577802; *Psittacine aviadenovirus C* (PsAdV-C), GenBank Ac No. MH580295 and crane-associated adenovirus 1 (CrAdV-1), GenBank Ac No. LC469780); they were aligned with MAFTT (version 7.388) [21] in Geneious Prime^®^ (version 2022.1.1, Biomatters, New Zealand). Conserved adenovirus genes, genus common and species-specific hypothetical ORFs and genes shared by both avidenovirus and mastadenoviruses were presented in different colours, as per the given guide. Conserved genes that are not present in genomes are marked with X, and genes present but not annotated are represented with a star (*). F-1 and F-2 are the abbreviations for fibre 1 and 2, respectively.

Aviadenoviruses possess a common adenovirus structural morphology, but with some notable distinctions. The fibre genes are a notable feature in the conserved region of the aviadenovirus genome, which is considered as a demarcation criterion in genome organization amongst aviadenovirus species [17]. The presence of two fibre genes is common within the genus *Aviadenovirus*; however, significant exceptions include the fowl aviadenovirus-B/D/E, turkey avidenovirus-B/C, crane-associated adenovirus 1, and duck avidenovirus B (Figure 5). However, the size and the protein products of their two fibre genes vary between species [17,18,46,56]. For example, the two fibre genes in *Pigeon aviadenovirus A* (GenBank Ac no. FN824512.2) encode 246aa and 749aa protein products and its closest relative aviadenovirus B (GenBank Ac no. YP_009310446.1) produces small and large fibre proteins with 147aa and 553aa, respectively. Additionally, some adenoviruses such as *Turkey aviadenovirus B* (GenBank Ac no. NC_014564) that encode two fibre genes have only one functional gene akin to aviadenovirus with a single fibre gene [63]. Fibre proteins are responsible for the efficient binding of the virus to its cellular receptor, and it is believed that the tropism of the adenovirus is determined by the presence of different fibre proteins [17,46]. A recent study on fowl adenovirus 4 (FAdV-4) found that even though fibre 2 is essential for replication, and assembly, and played a role in the virulence of the virus, fibre 1 is the key protein for direct mediation of the FAdV-4 infection via its knob and shaft domains [64]. Differences in the core genome have also been observed among individuals in the genus *Aviadenovirus*, such as the 33K protein-encoding gene being absent in *Psittacine aviadenovirus C* (Figure 5).

Aviadenoviruses are distinguished by having a comparatively higher number of novel genes in the right genomic end as well as the left-hand end of the genome. The genus-specific dUTP pyrophosphate (dUTPase) gene is situated in the left-hand end of the genome in the aviadenoviruses, whereas in some mastadenoviruses, the dUTPase gene is within the right genomic end. Its neighbouring NS1 and three other hypothetical protein-coding genes are common to all aviadenoviruses but multiple ORFs are found to be species-specific (Figure 5). However, the functionality of these novel genes remains unknown. 

Comparative analysis of G + C content at the genomic level and the pairwise analysis against other avian adenoviruses demonstrated a high genetic diversity amongst aviadenoviruses [10,15,17,33,42,65]. Compositions are quite similar in strains of fowl adenovirus with 53.8–57.9% of G + C; however, *Goose adenovirus A*, *Pigeon adenovirusA*, and *Turkey adenovirus B* have significantly different G + C contents, with 44.7%, 63.8%, and 66.9%, respectively [17,18,33] (Figure 3). Furthermore, a surprisingly low level of G + C content (34%) has been revealed in a nearly complete genome sequence of a new aviadenovirus, crane-associated adenovirus 1 [34].

#### 3.3.2. Evolution

The genus *Aviadenovirus* was named to represent the dominant avian hosts in it and is believed to be avian specific and to have co-evolved with birds [8]. However, as consistent with the *Atadenovirus* and *Siadenovirus* genera, the evolution of the *Aviadenovirus* genus also reflects a combination of host switching and virus–host coevolution. Avidenoviruses may have reflected coevolution with avian hosts [8], such as goose aviadenoviruses with their analogous *Galliformes* and that of fowl adenoviruses in birds belonging to the orders *Galliformes* and *Anseriformes*, but the aviadenovirus coevolution in avian hosts is not fully explained [18]. Similarly, aviadenoviruses originated from other wild birds, such as passerines and psittacines, which dominate in lineages 2 and 4 of the adenoviruses, respectively (Figure 3) supporting the phenomenon of aviadenovirus coevolution by grouping into a common clade, but this does not explain the appearance of a diverse range of bird hosts in the group (Figure 2 and Figure 3). 

Therefore, even though it is thought that host switches are somewhat infrequent amongst aviadenoviruses [30], co-speciation does not fully explain the aviadenovirus evolution, and several significant interclass host switches were identified during the evolution. The unexpected appearance of some viruses in the phylogenetic analysis demonstrated several possible host switching events (Figure 3) [33]. In the phylogenetic tree based on available aviadenovirus DNA polymerase sequences (Figure 2), a separate clade was formed by the crane-associated adenovirus 1 first infecting *Gruiformes*, which then evolved to host *Anseriformes*, *Psittaciformes*, and later to the *Galliformes*. It can be hypothesised that the coevolution of viruses with their host can possibly have a comparable evolutionary history. However, when considering the aviadenovirus host phylogeny, this is unlikely, as *Anseriformes* and *Galliformes* evolved in the early stage of bird evolution and *Psittaciformes* quite recently [66,67]. 

As shown in Figure 3, the unusual appearance of barn owl and boobook owl in distinct lineages (lineages 1 and 3), silver gull viruses in different lineages (1, 2, and 3), and Smooth-billed ani, which is a Brazilian tropical *Cuculiform* found in Brazil [43] appearing in lineage 1 (Figure 3), can be explained by possible virus host switching events. Aviadenoviruses detected in both European and Australian passerines might be due to the co-speciation of passerine virus followed by a possible host transmission with introduced European goldfinch to endemic Australian goldfinch. 

The recent detection of aviadenovirus in pine martens [68] and the proximity of marten and passerine adenovirus clades in the phylogeny (Figure 3) might not be a coincidence, as pine marten has the potential to acquire avian infections from a passerine. According to a study on pine marten diet composition, it is found that they fed on pigeons, starlings, and some passerines such as goldcrests [32], and the detection of aviadenovirus in marten could be a result of cross-species virus transmission [68]. Importantly, a recent discovery of a mastadenovirus sequence from a group of bird species (Figure 3) highlights an unprecedented event of adenovirus emergence [10]. The detected sequence was a variant of murine adenovirus 2 and was found in droppings of two galahs and two tawny frogmouths. The authors of that study assumed that these viruses could have possibly been transmitted via ingested materials and passed through the digestive tract, as galahs feed on the ground and could ingest food items contaminated with mouse faeces or urine, and tawny frogmouths feed on mice. However, the detection of the same sequence in the kidney of a sulphur-crested cockatoo and a boobook owl suggests that this mastadenovirus was replicating in these birds, and they were less likely infected by contaminated food. As shown in Figure 3, the identified murine adenoviruses 2 from bird species have developed a distantly related clade compared to other mastadenovirus members in the group (Figure 3). These findings warrant further investigation to understand the role of these bird species in the dissemination of mastadenoviruses, and further analysis of complete genomes of aviadenovirus is necessary for a clearer understanding.

## 4. Conclusions and Future Recommendation

Adenoviruses are a diverse group of viruses causing predominantly asymptomatic infections in almost every class of vertebrates. The recent proliferation of sequencing and bioinformatics analysis has expanded our understanding of adenovirus genomes and variations found therein. Amidst the fairly conserved core gene composition and arrangement, some adenovirus genomes lack a few core genes and/or genus-specific genes. Additionally, adenovirus genomes are highly divergent, with a varied number of hypothetical protein-coding genes in genome ends that contribute to the increased species diversity. Some of these ORFs are common within the genus or shared by a few genera, but further functional analysis is required to understand these variations at the genomic level. 

Until recently, very little sequence data was available for adenovirus infections in the wild, as studies mostly focused on human and other economically important adenovirus infections. Recently identified novel adenoviruses have demonstrated the significance of wild avian host species, as these wild hosts display varying feeding, roosting, and migration habitats which may enhance adenovirus diversity, as avian hosts might assist viruses in gaining new gene combinations and overcome genetic barriers to infection in new host species. Complete genome architectures and partial DNA polymerase sequences of adenoviruses uncovered from wild avian and reptile hosts in Australia and Europe from recent studies evidenced the notably higher adenovirus diversity and their radical evolutionary relationships [10,15,16,36,44,60,69,70]. The recent expansion of high throughput sequencing data and robust phylogenetic reconstructions emphasise the need for alterations to previous conclusions, including the reptilian origin of atadenoviruses and sole aviadenovirus infection on birds. Furthermore, a focus on wild avian species for novel adenovirus identification and their genomic characteristics with complete genome sequences will be necessary to clear the conflicting ideas on siadenovirus origin [8] and better understand the adenovirus evolution and distribution.

## Data Availability

Not applicable.

## Supplementary Materials

The following are available online at https://www.mdpi.com/article/10.3390/v14081767/s1, Table S1: Details of sequences used in the Tanglegram; Table S2: Similarity (%) across five different genera using amino acids sequences of selected complete DNA pol gene. Blosum62 with threshold 1 (percentage of residues which have score ≥ 1 in the Blosum62 matrix) parameter was chosen in Geneious Prime^®^ (version 2022.1.1, Biomatters, New Zealand) to calculate % similarity; Table S3: Details of sequences used in the phylogenetic tree; Figure S1: Phylogenetic tree was generated using concatenated amino acid sequences of four genes (DNA polymerase, penton, hexon, and pTP), Figure S2; Phylogenetic tree was generated using adenovirus DNA polymerase, and Figure S3; Phylogenetic tree was generated using Cytochrome c oxidase 1 subunit of representative hosts.
