# Supplementary material for: Adenoviruses in Avian Hosts: Recent Discoveries Shed New Light on Adenovirus Diversity and Evolution
Source: Viruses. 2022 Aug 13;14(8):1767. doi: 10.3390/v14081767 (PMC9416666; doi:10.3390/v14081767)
Supplement: Supplementary file 1 [file viruses-14-01767-s001.zip › Supplimentary Materials.pdf]

viruses

Article

Adenoviruses in Avian Hosts: Recent Discoveries Shed New Light on Adenovirus Diversity and Evolution

Ajani Athukorala[1], Karla J. Helbig[1], Brian P. Mcsharry[2], Jade K. Forwood[2] and Subir Sarker[1]*

[1] Department of Microbiology, Anatomy, Physiology and Pharmacology, School of Life Sciences, La Trobe University, Melbourne, VIC 3086, Australia
[2] School of Dentistry and Medical Sciences Biomedical Sciences, Charles Sturt University, Wagga Wagga, New South Wales, Australia

*Correspondence: s.sarker@latrobe.edu.au; Tel.: +61-3-9479-2317; Fax: +61-3-9479-1222

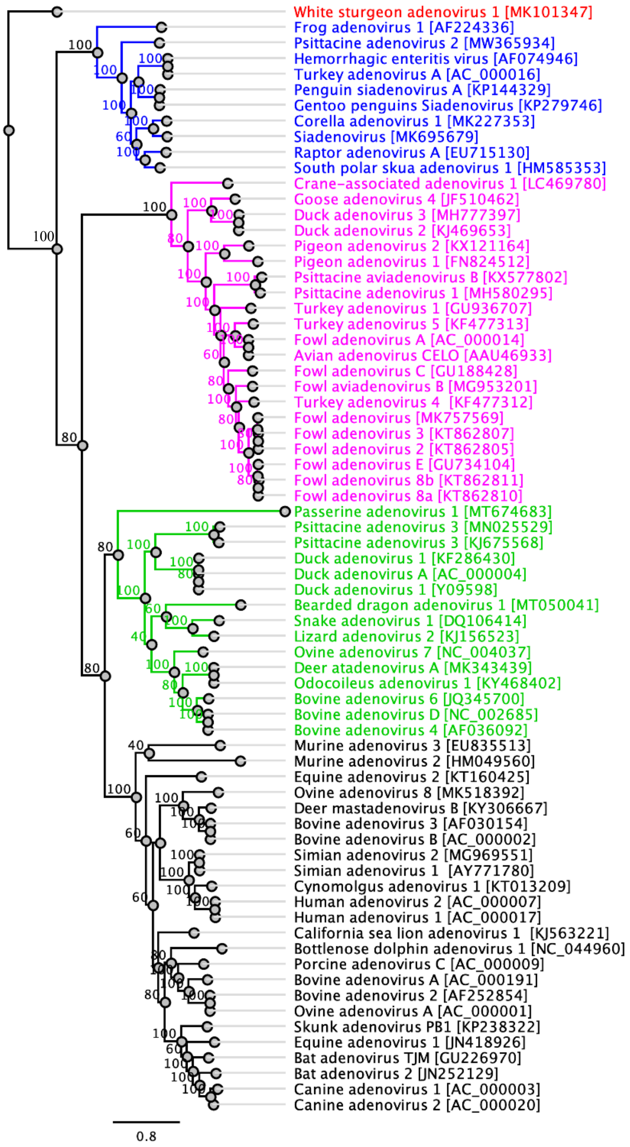

Figure S1. Maximum likelihood (ML) tree was generated using concatenated amino acid sequences of four major structural and functional genes; complete DNA-dependent DNA polymerase, penton, pTP and hexon. Concatenated protein sequences were aligned with MAFTT (version 7.388)[1] in Geneious (version 21.1.1) under the BLOSUM62 scoring matrix, and gap open penalty =1.53. The gap >30 residues were deleted from the alignments. The unrooted ML tree was constructed using the WAG substitution model with 1000 bootstrap re-samplings in Geneious (version 21.1.1). The numbers on the left show bootstrap values as percentages. The labels at branch tips refer to the original AdVs species name followed by the GenBank accession number in parentheses. The five official genera were highlighted as different background colours.

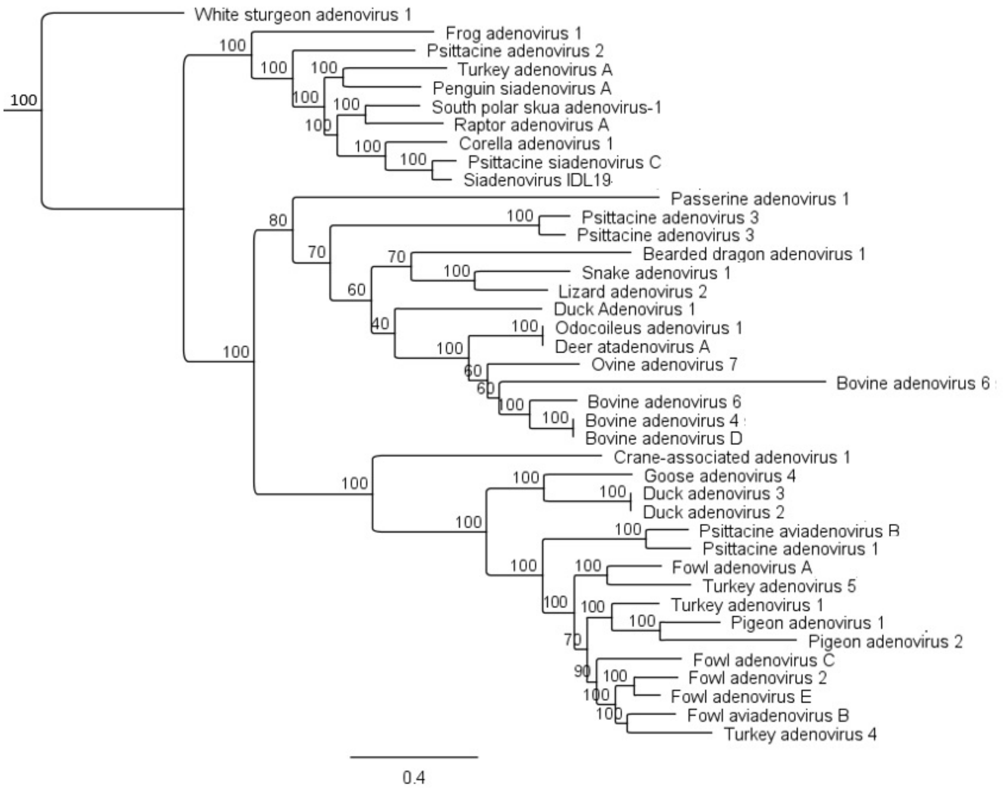

Figure S2. Maximum likelihood (ML) tree was generated with DNA polymerase gene sequence of selected adenoviruses (Supplementary Table S1). The unrooted ML tree was constructed with PhyML in Geneious (version 21.1.1) and 100 bootstrap re-samplings was chosen. The developed ML tree was then used in generating the tanglegram in Dendroscope (Version 3.8.2). The numbers on the left show bootstrap support as percentages.

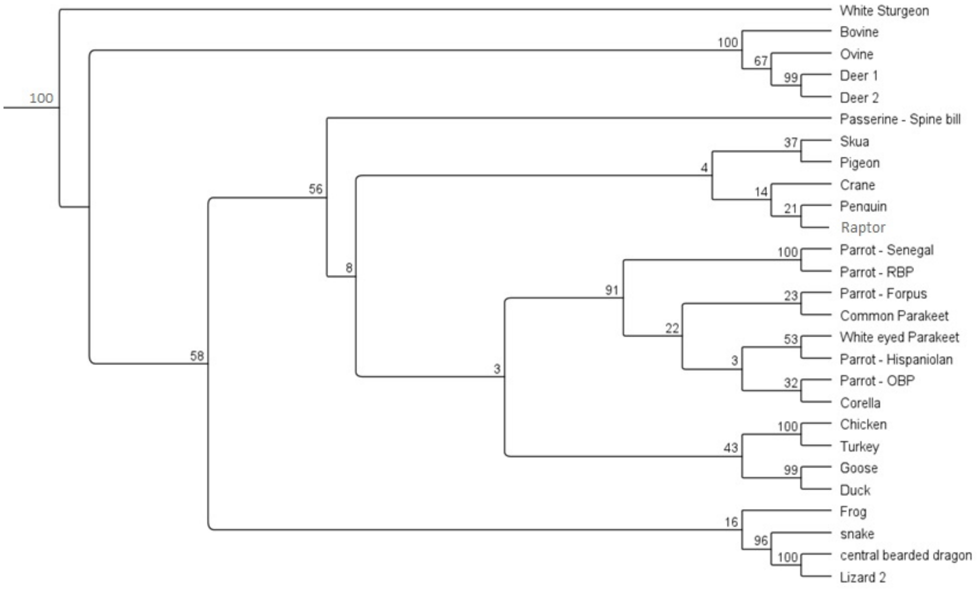

Figure S3. Maximum likelihood (ML) tree was generated with Cytochrome c oxidase 1 sequence of host organisms representing selected adenoviruses (Supplementary Table S1). The unrooted ML tree was constructed with PhyML in Geneious (version 21.1.1) and 100 bootstrap re-samplings was chosen. The developed ML tree was then used in generating the tanglegram in Dendroscope (Version 3.8.2). The numbers on the left show bootstrap support as percentages.

Table S1: Details of sequences used in the Tanglegram (Figure 2).

**Virus species**	**GenBank accession number of viruses**	**host species**	**host common name used in the figure**	**GenBank accession number of host**
Psittacine siadenovirus 2	MW365934	*Neophema chrysogaster*	Orange-bellied parrot	NC_019804.1
Penguin siadenovirus A	KP144329	*Pygoscelis antarcticus*	chinstrap penguin	NC_021474.1
Turkey adenovirus A	AC_000016	*Meleagris*	Turkey	EF153719.1
South polar skua adenovirus-1	HM585353	*Stercorarius maccormicki*	South polar skua	NC_026125.1
Raptor adenovirus A	EU715130	*Parabuteo unicinctus*	Raptor	NC057089.1
Corella adenovirus 1	MK227353	*Cacatua sanguinea*	Little Corella	MN126573.1
Psittacine siadenovirus C	MN687905	*Melopsittacus undulatus*	Common parakeet	KP137624.1
Siadenovirus IDL19-3602	MK695679	*Forpus coelestis*	Pacific parrotlet	NC_027843.1
passerine adenovirus 1	MT674683	*Acanthorhynchus tenuirostris*	Eastern spinebill	NC_051552.1
Psittacine adenovirus 3	MN025529	*Psittacara leucophthalmus*	White-eyed parakeet	NC_041257.1
Psittacine adenovirus 3	KJ675568	*Amazona farinosa*	Hispaniolan parrot	KX925977.1
Duck adenovirus 1	KF286430	*Muscovy duck (Cairina moschata)*	Duck	NC_010965.1
Crane-associated adenovirus 1	LC469780	*Gruidae*	Crane	NC_021368.1
Pigeon adenovirus 1	FN824512	*Columba*	pigeon	NC_013978.1
Psittacine adenovirus 1	MH580295	*Poicephalus senegalus*	Senegal parrot	NC_044083.1
Turkey adenovirus 1	GU936707	*Meleagris gallopavo*	Turkey	EF153719.1
Fowl aviadenovirus B	MG953201	*Gallus gallus domesticus*	Chicken	KX987152.1
Fowl aviadenovirus C	GU188428	*Gallus gallus domesticus*	Chicken	KX987152.1
Goose adenovirus 4	JF510462	*Anser anser*	Goose	MN122908.1
Duck adenovirus 2	KJ469653	*Muscovy duck (Cairina moschata)*	Muscovy duck	NC_010965.1
Deer atadenovirus A	MK343439	*Cervus canadensis*	Deer	JN632670.1
White Sturgen adenovirus 1	MK101347	*Acipenser transmontanus*	White Sturgeon	NC_004743.1
Bearded dragon adenovirus 1	MT050041	*Pogona vitticeps*	Bearded dragon	MZ59532
Snake adenovirus 1	DQ106414	*Pantherophis slowinskii*	Snake	DQ523162.1
Lizard adenovirus 2	KJ156523	*Heloderma horridum*	Lizard 2	KU986270
Bovine adenovirus 4	AF036092	*Bos taurus*	cattle	GU947021.1
Bovine adenovirus 6	JQ345700	*Bos taurus*	cattle	GU947021.1
Odocoileus adenovirus 1	KY468402	*Odocoileus hemionus*	Mule deer	JN632670.1
Ovine adenovirus 7	NC_004037	*Ovis aries*	sheep	MW364895.1
Fowl aviadenovirus E	GU734104	*Gallus gallus domesticus*	Chicken	KX987152.1
Frog adenovirus 1	AF224336	*Rana temporaria*	Frog	NC_042226.1

Table S3: Details of sequences used in the phylogenetic tree.

**Adenovirus name**	**Abbreviation used in the phylogram**	**GenBank accession number**
Atadenovirus	vitelline 1	MN380538
Atadenovirus	vitelline 2	MN380539
Atadenovirus	vitelline 5	MN380542
Atadenovirus	vitelline 6	MN380543
Atadenovirus	song thrush	MN380548
Atadenovirus	European greenfinch 1	MN380551
Atadenovirus	European robin	MN380552
Aviadenovirus	vitelline 3	MN380540
Aviadenovirus	vitelline 4	MN380541
Aviadenovirus	vitelline 7	MN380544
Aviadenovirus	great tit 1	MN380547
Aviadenovirus	European goldfinch	MN380553
Aviadenovirus	European greenfinch 2	MN380554
Siadenovirus	zebra finch 1	MN380537
Siadenovirus	zebra finch 2	MN380545
Siadenovirus	zebra finch 3	MN380546
Siadenovirus	zebra finch 2	MN380549
Siadenovirus	barred finch	MN380550
Siadenovirus	great tit 2	MN380555
Siadenovirus	Eurasian blackcap	MN380556
Siadenovirus	great tit 3	MN380557
Siadenovirus	Eurasian bullfinch	MN380558
Siadenovirus	common chaffinch	MN380559
Siadenovirus	Eurasian siskin	MN380560
Siadenovirus	zebra finch 4	MN380561
Smooth billed ani adenovirus 1	smooth-billed	MN540448
Tropical screech owl adenovirus 1	tropical screech owl	MN540447
Agamid adenovirus 1	agamid	FJ196812.1
Amniota adenovirus	amniota	MN025530
Amphisbaenian adenovirus 1	amphisbaenian	KT950887.1
Anolis sagrei adenovirus1	anolis 1	MH558562.1
Anolis sagrei adenovirus 2	anolis 2	MH558564.1
Anolis sagrei adenovirus 3	anolis 3	MH558565.1
Anolis sagrei adenovirus 4	anolis 4	MH558563.1
Anolis sagrei adenovirus 5	anolis 5	MH558569.1
Anolis sagrei adenovirus 6	anolis 6	MH558568.1
Anolis sagrei adenovirus 7	anolis 7	MH558567.1

**Adenovirus name**	**Abbreviation used in the phylogram**	**GenBank accession number**
Anolis sagrei adenovirus 8	anolis 8	MH558566.1
Bat adenovirus TMJ	bat	GU226970.2
Bovine adenovirus 4	bovine 4	AF036092.3
Bovine adenovirus 1	bovine 1	NC_044934
California sea lion adenovirus 1	sea lion	KJ563221.1
Chameleon adenovirus 1	chameleon	AY576679.1
Canine adenovirus type 1	canine 1	U55001.1
Duck atadenovirus A	duck A	AC_000001
Estrildidae adenovirus 1	estrildidae 1	MK413652.1
Estrildidae adenovirus 2	estrildidae 2	MK413651.1
Eublepharid adenovirus 1	eublepharid	AY576677
Fowl aviadenovirus A	fowl A	AC_000014.1
Fowl adenovirus 5	fowl 5	KC493646.1
Fowl aviadenovirus C	fowl C	GU188428.1
Fowl aviadenovirus 2	fowl 2	HM853995.1
Fowl adenovirus 6	fowl 6	KT862808.1
Frog adenovirus 1	frog	NC_002501.1
Gekkonid adenovirus 1	gekkonid	AY576681.1
Goose adenovirus 4	goose 4	JF510462.1
Gouldian finch adenovirus 1	g. finch	KF031569.1
Great tit siadenovirus A	great tit	FJ849795.1
Gull adenovirus	gull	KC309438.1
Helodermatid adenovirus 1	helodermatid	AY576680.1
Human mastadenovirus A	human A	AC_000005.1
Kowari adenovirus	kowari	KT696557.1
Lacertid adenovirus 1	lacertid 1	KT950888.1
Lacertid adenovirus 2	lacertid 2	KT950885.1
Little corella adenovirus	l. corella	MK227353.1
Lizard adenovirus 2	lizard 2	KJ156523.1
Marten adenovirus	marten	KY705358.1
Meyer's parrot adenovirus 1	Meyer's parrot	AY644731.1
Murine adenovirus 1	murine 1	AC_000012.1
Murine adenovirus 2	murine 2	HM049560.1
Murine adenovirus 3	murine 3	EU835513.1
Odocoileus adenovirus A	deer A	NC_035619.1
Ovine adenovirus 7	ovine 7	U40839.3
Ovine adenovirus A	ovine A	AC_000001.1
Pancake tortoise adenovirus	pancake tortoise	JN632575.1
Penguin siadenovirus A	penguin	KP144329.1
Pigeon adenovirus 1	pigeon 1	FN824512.1

Adenovirus name	Abbreviation used in the phylogram	GenBank accession number
Pigeon adenovirus 2	pigeon 2	KX121164.1
Pigeon adenovirus 4	pigeon 4	KX555531.1
Pigeon adenovirus 5	pigeon 5	KX555532.1
Psittacine adenovirus 3	psittacine 3	KJ675568.1
Psittacine 3 type 1	psittacine 3 type 1	MN025529.1
Psittacine adenovirus 2	psittacine 2	EU056825.1
Psittacine aviadenovirus B	psittacine B	KX577802.1
Psittacine adenovirus 1	psittacine 1	MH580295.1
Psittacine siadenovirus D (proposed name)	psittacine D	MN687905
Raptor adenovirus 1	raptor 1	NC_015455.1
Red-eared slider adenovirus	red-eared slider	JN632580.1
Scincid adenovirus 1	scincid 1	AY576682.1
Siadenovirus IDL-3602	pacific parrotlet	MK695679
Simian adenovirus 3	simian 3	AY598782.1
Skunk adenovirus PB1	skunk	KP238322.1
South polar skua adenovirus-1	skua 1	HM585353.1
Snake adenovirus 2	snake 2	FJ012163.1
Snake adenovirus 3	snake 3	FJ012164.1
Southern Psittacara leucophthalmus aviadenovirus 1	white-eyed parrot	MN153802.1
Turkey adenovirus 1	turkey 1	GU936707.2
Turkey siadenovirus A	turkey A	AC_000016.1
Western bearded dragon Adenovirus 1	bearded Dragon	HQ005514.1
Yellow-bellied slider adenovirus	yellow-bellied slider	JN632578.1
